# Predictive value of a novel digital risk calculator to determine early patient outcomes after major surgery: a proof-of-concept pilot study

**DOI:** 10.1186/s13037-024-00395-y

**Published:** 2024-04-12

**Authors:** Svenja Sliwinski, Sara Fatima Faqar-Uz-Zaman, Jan Heil, Lisa Mohr, Charlotte Detemble, Julia Dreilich, Dora Zmuc, Wolf O. Bechstein, Sven Becker, Felix Chun, Wojciech Derwich, Waldemar Schreiner, Christine Solbach, Johannes Fleckenstein, Natalie Filmann, Andreas A. Schnitzbauer

**Affiliations:** 1 Department for General, Visceral, Transplant and Thoracic Surgery, Frankfurt University Hospital, Goethe University Frankfurt/Main, Theodor-Stern-Kai 7, 60590 Frankfurt/Main, Germany; 2 Institute of Sports Medicine, Goethe University Frankfurt/Main, Frankfurt/Main, Germany; 3 Department for Gynecology, Frankfurt University Hospital, Goethe University Frankfurt/Main, Frankfurt/Main, Germany; 4 Department for Urology, Frankfurt University Hospital, Goethe University Frankfurt/Main, Frankfurt/Main, Germany; 5 Department for Vascular Surgery, Frankfurt University Hospital, Goethe University Frankfurt/Main, Frankfurt/Main, Germany; 6 Pain Center, Hospital Landsberg am Lech, Landsberg am Lech, Germany; 7 Institute of Biostatistics and Mathematical Modeling, Goethe-University Frankfurt, Frankfurt/Main, Germany

**Keywords:** Prehabilitation, Frailty, Postoperative complications, App, Risk score

## Abstract

**Background:**

A structured risk assessment of patients with validated and evidence-based tools can help to identify modifiable factors before major surgeries. The Protego Maxima trial investigated the value of a new digitized risk assessment tool that combines tools which can be easily used and implemented in the clinical workflow by doctors and qualified medical staff. The hypothesis was that the structured assessment and risk-grouping is predictive of short-term surgical quality reflected by complications and overall survival.

**Methods:**

The Protego Maxima Trial was a prospective cohort analysis of patients undergoing major surgery (visceral, thoracic, urology, vascular and gynecologic surgeries) as key inclusion criterion and the absence of an acute or acute on chronically decompensated pulmo-cardiovascular decompensation. Patients were risk-scored with the software (The Prehab App) that includes a battery of evidence-based risk assessment tools that allow a structured risk assessment. The data were grouped to predefined high and low risk groups and aggregate and individual scores. The primary outcome was to validate the predictive value of the RAI score and the TUG for overall survival in the high and low risk groups. Secondary outcomes were surgical outcomes at 90-days after surgery (overall survival, Clavien-Dindo (CD) 1–5 (all complications), and CD 3–5 (major complications)). The study was carried out in accordance with the DIN ISO 14,155, and the medical device regulation (MDR) at Frankfurt University Hospital between March 2022 and January 2023.

**Results:**

In total 267 patients were included in the intention to treat analysis. The mean age was 62.1 ± 12.4 years. Patients with a RAI score > 25 and/or a timed up and go (TUG) > 8 s had a higher risk for mortality at 90 days after surgery. The low-risk group predicted beneficial outcome and the high-risk group predicted adverse outcome in the ROC analysis (Area Under the Curve Receiver Operator Characteristics: AUROC > 0.800; *p* = 0.01). Risk groups (high vs. low) showed significant differences for 90-day survival (99.4% vs. 95.5%; *p* = 0.04) and major complications (16.4% vs. 32.4%; *p* < 0.001).

**Conclusion:**

The proof-of-concept trial showed that a risk assessment with ‘The Prehab App’ may be viable to estimate the preoperative risk for mortality and major complications before major surgeries. The overall performance in this initial set of data indicated a certain reliability of the scoring and risk grouping, especially of the RAI score and the TUG. A larger data set will be required to proof the generalizability of the risk scoring to every subgroup and may be fostered by artificial intelligence approaches.

**Trial registration:**

Ethics number: 2021-483-MDR/MPDG-zuständig monocentric; The Federal Institute for Pharmaceuticals and Medical Devices/BfArM, reference number: 94.1.04-5660-13655; Eudamed: CIV-21-07-0307311; German Clinical Trial Registry: DRKS 00026985.

**Supplementary Information:**

The online version contains supplementary material available at 10.1186/s13037-024-00395-y.

## Background

Major surgery is associated with a high rate of postoperative complications and reaches numbers as high as 15–40% [1, 2]. Almost half of all adverse events in hospitalized patients are related to the surgical procedure, morbidity, mortality, length of hospital stay, and healthcare costs in general [3]. Several studies have determined predictors of high complication rates using validated assessment tools, including the American Society of Anesthesiologists (ASA) Physical Status Classification, the Eastern Cooperative Oncology Group (ECOG) Performance Status, the Timed Up and Go-Test (TUG), and the Risk Analysis Index (RAI-score) [4–8].

The advantage of knowing the risk factors before surgeries are as simple as practical: modifiable factors can potentially be improved by an approach called prehabilitation, and unmodifiable factors help to weigh the risk of surgery against the benefit. Today, the average age of cancer diagnosis is 65 years, and more than 70% of early solid organ cancers can be cured with surgery [9, 10]. Thus, it is important to identify the patients at risk with validated and evidence-based tools, as mentioned above.

In general, frail patients are the major target for a risk assessment. However, frailty is present in the vast majority of older adults > 65 years, as recently shown by a Chinese group, and affects practically all octogenarians and almost 60% of the 65-79-year-old general population, which allows the assumption that frailty has a highly probable underreporting in the younger population, too [11]. Consequently, practically every patient can be improved before a surgical procedure. And honestly, who would run a marathon without adequately preparing for it? And surgery in this context is comparable with a marathon.

Prehabilitation, an emerging field in perioperative medicine defined by the implementation of proactive, exercise-based interventions to increase patient preparedness in the lead-up to surgery, may be a critical improvement for the operability of such patients [12, 13]. Interventions may include aerobic exercise, resistance or functional training, nutritional supplementation, and psychological interventions to optimize the patient’s functional capacity before surgery, reducing surgery-related morbidity and facilitating recovery after surgery [14]. It has been shown that prehabilitation reduces morbidity by up to 50% and overall costs by up to 30% and generates benefits for all stakeholders in the healthcare system [15–17].

Digital therapeutics in this context may be supportive and well-received by the patients and the doctors for several reasons: they can be well-integrated into the hospital workflow, save time, and deliver a high-quality set of structured data. These data can be used to generate exercise programs based on the individual patient risk algorithmically. They can furthermore help with individual dashboarding on a patient, center-level, or benchmarking against single indications or complete ecosystems [8]. In this prospective uncontrolled clinical cohort trial, a new medical device under development (The Prehab App Doctor’s module) has been evaluated in its potential to digitally assess patient data and correlate the risk assessment with 90-day mortality and overall morbidity. We hypothesized that the risk assessment is highly associated with 90-day mortality.

## Methods

### Study design and patients

The Protego Maxima trial is an investigator-initiated, prospective, interventional pilot study to assess the feasibility and safety of ‘The Prehab App’ in patients undergoing elective major surgery at the University Hospital in Frankfurt, Germany. The study protocol complies with the European Norm (EN) German Institute for Standardization (DIN) International Standardization Organization (ISO) 14,155, the MDR (medical device regulation), and the German Medical Device Implementation Act (MPDG). Ethical approval for this study was granted by the independent review board (IRB) of the Medical Faculty, Goethe University Frankfurt/Main (2021-483-MDR/MPDG-zuständig monocentric) and the Federal Institute for Pharmaceuticals and Medical Devices (BfArM, reference number 94.1.04-5660-13655; Eudamed, CIV-21-07-0307311) on February 7, 2022. The study was registered in the German Clinical Trial Registry (DRKS 00026985) on December 21, 2021, and in the European database on medical devices (Eudamed, CIV-21-07-0307311). Funding was received by the Else Kroener Fresenius Foundation in the translational research program under the number: 2021_EKTP10 (https://www.ekfs.de/en/scientific-funding/currently-funded-projects/development-interactive-app The study consisted of a usability trial, and an additional safety and validity trial of the patient’s app that are reported elsewhere. Details are outlined in the study protocol for the tasks not published here [18]. The work has been reported in accordance with the Strengthening the Reporting of cohort, cross-sectional and case-control studies in Surgery (STROCSS) criteria for cohort trials [19]. Patients were recruited between March 8th, 2022 with first patient-first visit and October 31st, 2022, with the last patient-last visit taking place on January 31st, 2023. All patients were recruited at the Centre of surgery at Frankfurt University Hospital.

### Inclusion and exclusion criteria

Eligibility criteria were adult patients aged ≥ 18 years, participants able to understand the respective task and provide written informed consent, and patients undergoing one of the following elective major surgeries: gastrointestinal (GI) resection, resection of the hepatobiliary and pancreatic system (HPB), endocrine glands, lung or bronchus, splenectomy, abdominal wall hernia, urological or gynecological resection or vascular surgery without cardiovascular procedures. Exclusion criteria were anamnestic pregnant or breastfeeding patients, the inability to understand or participate in the task, acute cardiovascular or pulmonary disease, or acute non-cardiopulmonary disorders that might affect or be aggravated by exercise performance (e.g., infection, renal failure). Patients scheduled for elective major surgery were screened for eligibility by the study team either in the outpatient departments or the day before surgery. Postoperative complications were graded according to the Clavien-Dindo (CD) classification.

The eligible cohort patients undergoing elective major surgery were screened and risk-scored prospectively using The Prehab App. Results were correlated with the 90-day outcomes as the key objective of the clinical investigation. Therefore, a follow-up was performed as a structured telephone interview to assess complications according to CD and overall survival. The primary endpoint was the predictive potential of the RAI score and the TUG for mortality. Secondary outcome measures were the analysis of mortality, the overall complication rate at day 90, and the major complication rate in accordance with Clavien and Dindo after major surgical procedures. Psoas density measurements and their potential in the prediction of frailty were added in patients with an available CT or MRI scan as a potential quality control parameter. Uni- and multivariable analysis were performed to identify potential independent predictors for the outcomes.

### The prehab app

The Prehab App is developed as a medical device class IIa under the legislation of the MDR and all applicable legislations. The app includes clinically evaluated risk scores, which are usable without extra tools. A special algorithm uses the Karvonen method and the presence of heart rate-modifying medication to calculate an individual endurance interval training for the patient as a prehabilitation program [21, 22]. The structured risk assessment of the app is validated by correlating its data with complications according to complication rates following Clavien-Dindo [20, 21], including overall survival, indication, and diagnosis. The risk assessment comprised the following data: birth year, sex, height (cm), weight (kg), body mass index (BMI), smoking (yes/no), resting pulse (bpm), Eastern Cooperative Oncology Group (ECOG), Timed Up and Go Testing in seconds (TUG; sec.), hemoglobin (HgB; g/dl) and the Risk Analysis Index (RAI score). Diagnosis, and surgical procedure were determined using the International Classification of Diseases 10th Revision (ICD-10) and the Operation Procedures System (OPS) for surgical procedures. Psoas density measurements were caried out in a standardized fashion [22].

### Study procedure

Patients were screened and scored after informed consent. An investigator performed the scoring with ‘The Prehab App’. Data were stored on DIN ISO 27,001 servers in the European Union and transferred into the eCRF. The RAI score was automatically calculated within the app, as was the risk group. ICD 10 and OPS coding as well as complication data were extracted from the electronic health record at the University Hospital Frankfurt (completely digitized assessment), and 90 days after the surgical procedure a structured telephone interview was carried out with each individual patient to assess complications occurring between discharge and day 90 after surgery. Additionally, patient charts were screened for additional information. In case a patient died the data were obtained from their general practitioner or the hospital information system (in house death). In accordance with Clavien-Dindo scoring only the most severe complication per patient was documented, and a complication of 3 or higher was considered major [20]. The data were also entered in the App and stored on the same servers in the same database.

### Risk groups

Different risk groups were calculated based on the following definitions: TUG < 10 s = 0, TUG = 10–20 s = 1, TUG > 20 s = 2; ECOG 0 = 0, ECOG1/2 = 1, ECOG3 = 2; HgB ≥ 13 g/dl = 0, HgB < 13 g/dl = 2, RAI < 16 = 0, 16–30 = 1, > 30 = 2. The TUG + ECOG + RAI + HgB score sum of 0 and 1 defined low-risk patients, and a risk score sum of ≥ 2 defined high-risk patients [23–26].

Moreover, data on the prevalence of sarcopenic patients were collected from computer tomography (CT) or magnetic resonance imaging (MRI) scans and evaluated by density measurement around the lumbar vertebrae three as skeletal muscle area in [cm^2^] (SMA) and calculated as skeletal muscle index (SMI) by dividing SMA through height^2^ [cm^2^/m^2^]. A patient was defined sarcopenic if the following criteria were true: female and SMI < 41 cm^2^/m^2^; male + BMI < 25 and SMI < 43 cm^2^/m^2^; male + BMI > 25 and SMI < 53 cm^2^/m^2^ as published by [22].

### Sample size and power calculation

The areas under the curve (AUC) for the crude patients’ risk scores were calculated for all complications, for major complications and for mortality at day 90 after surgery and compared between the high-risk group and the low-risk group. To detect an AUC ≥ 0.75 (as the level of relevance with a 95% confidence interval length of at least 0.15) the sample size of the cohort must be not less than *N* = 82.

The study provided a power of 80% for the primary tasks, assuming a *P-*value of ≤ 0.05 as statistically significant [27]. The power calculations were conducted using PASS 2008 (Version 08.0.15, NCSS, LLC, Kaysville, Utah) and BiAS (Version 11.0, epsilon 2015).

### Statistical analysis

All statistical analyses were performed using IBM SPSS statistical software version 25 for Windows (IBM Corp., Armonk, New York, USA). Continuous variables are expressed as mean ± standard deviation (SD), and categorical variables are presented as frequency and percentage for demographic data. Categorical variables were compared using the Chi-square test. Univariate and multivariate logistic regression analyses were performed to identify risk factors associated with postoperative complications and mortality. The results are the hazard ratio (HR) with a 95% confidence interval (CI). A two-sided *p* < 0.05 was considered statistically significant. Areas under the curve (AUC) were calculated for risk scores and risk groups, and receiver operating curve (ROC) analyses were performed to obtain cut-off values for the RAI score and the TUG test. Kaplan-Meier survival analysis was calculated to compare risk groups.

## Results

### Study recruitment and inclusion

A total of 306 patients were screened between March and October 2022, of which 39 did not meet the inclusion criteria or violated the exclusion criteria during screening. All patients could be followed up until day 90 after surgery or death (within 90 days after surgery). The final cohort for analysis consisted of 267 patients undergoing major surgical procedures in abdominal, thoracic, vascular surgery, gynecology or urology [28]. 

### Baseline demographics

Eighty-seven (33.0%) females and 180 (67.0%) males were included in the trial. The mean age was 62.1 ± 12.4 years. The mean BMI was 26.5 ± 5.5, with 88 (33%) smokers. Only 8.2% (*n* = 22) received a prescription for protein-enriched nutrition from their treating doctor.

The mean preoperative hemoglobin was 13.5 ± 1.8 g/dl. Anemia before surgery was present in 89 patients (33%), of which 47 were female and 42 were male. The mean preoperative resting pulse was 74.7 ± 13.5 bpm with no gender-specific differences. The mean TUG-test time was 7.7 ± 2.3 s. The ECOG score was 0 in 89.1% (*n* = 238), 1 in 8.6% (*n* = 23), 2 in 1.9% (*n* = 5), and 3 in 0.4% (*n* = 1). The mean RAI score was 21.5 ± 9.7, with cancer being the leading diagnosis in 195 (73%) patients. Weight loss was present in 28.8%, chronic renal failure in 10.1%, chronic compensated cardiac failure in 4.5%, and inappetence in 13.5%. In contrast, shortness of breath was assessed in 0.7% of all patients as a risk factor. The RAI-scoring contains activities of daily living available before surgery for mobility, eating, toilet use, and self-care/personal hygiene in categories independent (0) to fully dependent [4], with most patients being full to majorly independent. The cognitive status had worsened three months before the planned surgery in 16 patients (6.0%). Patients were classified into two risk categories according to the presence of risk factors. Risk Group 1 (low risk) comprised 156 patients (58.4%). Risk Group 2 (high risk) included 111 (41.6%) patients. 82 (72.5%) out of 113 patients with available CT or MRI scans were defined as sarcopenic. Data are displayed in Table 1.

**Table 1:** Demographic and risk data in the screened cohort of patients. §§: TUG < 10 = 0, TUG10-20 = 1, TUG > 20 = 2; ECOG 0 = 0.ECOG1/2 = 1, ECOG3 = 2; HgB < 13 = 2, RAI < 16 = 0, 16–30 = 1, > 30 = 2 Risk score Sum 0 and 1: low risk, ≥ 2: high risk. Abbreviations: y: yes; f: female; m: male; HgB: hemoglobin bpm: beats per minute; SMA: skeletal muscle area; SMI: skeletal muscle index; ECOG: Eastern Cooperative Oncology Group; RAI: risk analysis index; ADL: activities of daily living; n: number

Baseline demographics and risk factors	Subcategory	Total *n* = 267
Age (y)		62.1 ± 12.4
Sex (f/m)		
	Female	87 (33.0%)
	Male	180 (67.0%
BMI		26.5 ± 5.5
Smoker		88 (33%)
Protein-enriched nutrition (Recommended by doctor; y (%)		22 (8.2%)
Preoperative Hgb (g/dl)		13.5 ± 1.8
Anemia (y (%))		89 (33%)
Preoperative Resting Pulse (bpM)		74.7 ± 13.5
SMA [cm^2^]		116.2 ± 35.9
SMI [cm^2^/m^2^]		38.4 ± 9.4
Sarcopenia (y (%))		82 (72.5%)
ECOG	0 1 2 3	238 (89.1%) 23 (8.6%) 5 (1.9%) 1 (0.4%)
TUG (sec.)		7.7 ± 2.3
RAI score		21.5 ± 9.7
	Cancer (y (%))	195 (73.0%)
	Weight loss (y (%))	77 (28.8%)
	Renal failure (y (%))	27 (10.1%)
	Cardiac failure (y (%))	12 (4.5%)
	Inappetence (y (%))	36 (13.5%)
	Shortness of breath (y (%))	2 (0.7%)
ADL mobility	0| 1| 2| 3| 4	255| 3| 2| 3| 4
ADL eating	0| 1| 2| 3| 4	257| 2| 2| 1| 5
ADL toilet use	0| 1| 2| 3| 4	259| 0| 2| 1| 5
ADL hygiene/self-care	0| 1| 2| 3| 4	255| 3| 2| 3| 4
	Cognitive Status Worsened (y (%))	16 (6.0%)
Risk category of patients^§§^	0/1 (low risk)	156 (58.4%)
	> 2 (high risk)	111 (41.6%)
Medical Specialty	Visceral Surgery	111 (41.6%)
	Thoracic Surgery	39 (14.6%)
	Urology	80 (30.0%)
	Gynecology	15 (5.6%)*
	Vascular Surgery	22 (8.2%)

### Specialties, surgical procedures, complications, and 90-day mortality

Patients from different surgical specialties were included. 41.6% from the Department of Visceral Surgery (*n* = 111), 14.6% from the Department of Thoracic Surgery (*n* = 39), 30.0% from the Department of Urology (80), 5.6% from the Department of Gynecology (*n* = 15), and 9.2% (*n* = 22) were patients undergoing vascular surgery. The most prevalent major surgical procedures in urology were prostatectomies (22.5%, *n* = 60), nephrectomies (2.6%, *n* = 7), and others (4,9%, *n* = 13). Ovariectomies (2.2%, *n* = 6), hysterectomies (1.1%, *n* = 3), and others (4.1%, *n* = 11) were most prevalent in the Gynecologic Department. Upper GI surgeries (5.6%, *n* = 15), followed by liver and biliary resections (12.7%, *n* = 34), pancreatectomies (3.4%, *n* = 9), resections of the small or larger bowel (6.7%), endocrine resections (4.9%, *n* = 13) and hernia repairs (6.4%, *n* = 17) were included from the visceral surgeons and surgical oncology. Thoracic surgical patients had lung resections (14.6%, *n* = 39), and in vascular surgery, procedures of the carotids, the aorta, and arteries of the legs (iliac and femoral) were performed (8.2%, *n* = 22). Complications according to the Clavien-Dindo grading were as follows: Category 0 in 40.1% (*n* = 107), category 1 in 6.7% (*n* = 18), category 2 in 30.7% (*n* = 82), category 3a in 9% (*n* = 24), category 3b in 8.2% (*n* = 22), category 4a in 2.2% (*n* = 6), category 4b in 0.7% (*n* = 2), category 5 in 2.2% (*n* = 6). Complication analysis found major complications (≥ CD 3a) in 22.5% (*n* = 60, CD 3 to 5). Overall complication rates (CD 1–5) occurred in 59.9% (*n* = 160) at 90 days. The 90-day mortality was 2.2% (*n* = 6). Notably, more than 90% of complications occurred during the hospital stay. Data are displayed in Table 2.

**Table 2:** operative management, complications, and outcome. Abbreviations: n: number; GI: gastrointestinal; *gynecologicprocedures were multi-visceral resections, including visceral surgery in 5 cases explaining the discrepancy between the five proceduresmore in gynecology when compared to the counts in a medical specialty

Operative management, complications, and mortality	Subcategory	Total *n* = 267
Major surgical procedure	Nephrectomy Prostatectomy Urology other Hysterectomy Ovarectomy Gynecology other* Upper GI Liver & Biliary Pancreatectomies Lower GI Endocrine Hernias Lung Resections Vascular Surgery	7 (2.6%) 60 (22.5%) 13 (4.9%) 3 (1.1%) 6 (2.2%) 11 (4.1%) 15 (5.6%) 34 (12.7%) 9 (3.4%) 18 (6.7%) 13 (4.9% 17 (6.4%) 39 (14.6%) 22 (8.2%)
Dindo-Clavien category of complications	0 1 2 3a 3b 4a 4b 5	107 (40.1%) 18 (6.7%) 82 (30.7%) 24 (9.0%) 22 (8.2%) 6 (2.2%) 2 (0.7%) 6 (2.2%)
Outcomes	Complications (DC 1 to 5) Major Complications (DC 3 to 5) 90-day Mortality	160 (59.9%) 60 (22.5%) 6 (2.2%)

### Complication rates compared between specialties and surgical procedures

A total of 107 patients did not have any complications after surgery (40.1%). Minor complications type CD I and II occurred in 100 patients (37.5%) and were equally distributed between all medical specialties (visceral: 43 out of 74; thoracic: 16 out of 25; urology: 29 out of 43; gynecology: 5 out of 6, and vascular surgeries seven minor complications out of 11 complications in total).

### Medical specialties

There were no differences in mortality rates. Three patients out of 111 died in the visceral group (2.7%), 1 out of 38 in the thoracic group (2.6%), 1 out of 79 (1.3%) in the urology group, and 1 out of 21 (4.7%) in the vascular group. In contrast, no death case was observed in the 15 gynecologic patients included (*p* = 0.85).

Overall complication rates (59.9%; *p* = 0.17) and major complication rates (22.5%; *p* = 0.27) were not different between the medical specialties, although the ratio of high-risk patients was the highest in visceral surgery (53%), followed by thoracic surgery (51%), vascular surgery (50%), gynecology (47%) and urology (18%).

### Surgical procedures

The complication rates between the surgical procedures were significantly different (*p* = 0.007), whereas the occurrence of the total numbers within the CD classes was not different between the procedures (*p* = 0.06). Pancreatic surgery (88%), Upper GI (87%), liver/biliary surgery (79%), lower GI surgery, kidney surgery (71%), and ovariectomies (67%) had the highest CD 1 to 5 complication rates, followed by thoracic surgery (64%), vascular surgery (54%), prostatectomies (53%), other urologic resections and endocrine surgeries (46%), nephrectomies (43%), other gynecologic resections (36%), and hysterectomies (0). Data for complications in medical specialties and specifically for each surgical procedure can be found in the supplementary tables and figures.

### Predictive risk factors for postoperative morbidity & mortality

ROC analyses were performed to analyse the potential of the composite risk score to predict outcomes. In brief, the ROC analysis showed a highly significant potential of the risk score in its prediction of mortality for the high-risk group (AUROC = 0.842) and for the low-risk group (AUROC = 0,990). There was a difference in AUROCs of -0.149 (95% CI: -0.263; -0.034) with a p-value of 0.01. Analysis for major complications and all complications occurring until day 90 after surgery were not predictive in the ROC analysis.

**Fig. 1:**
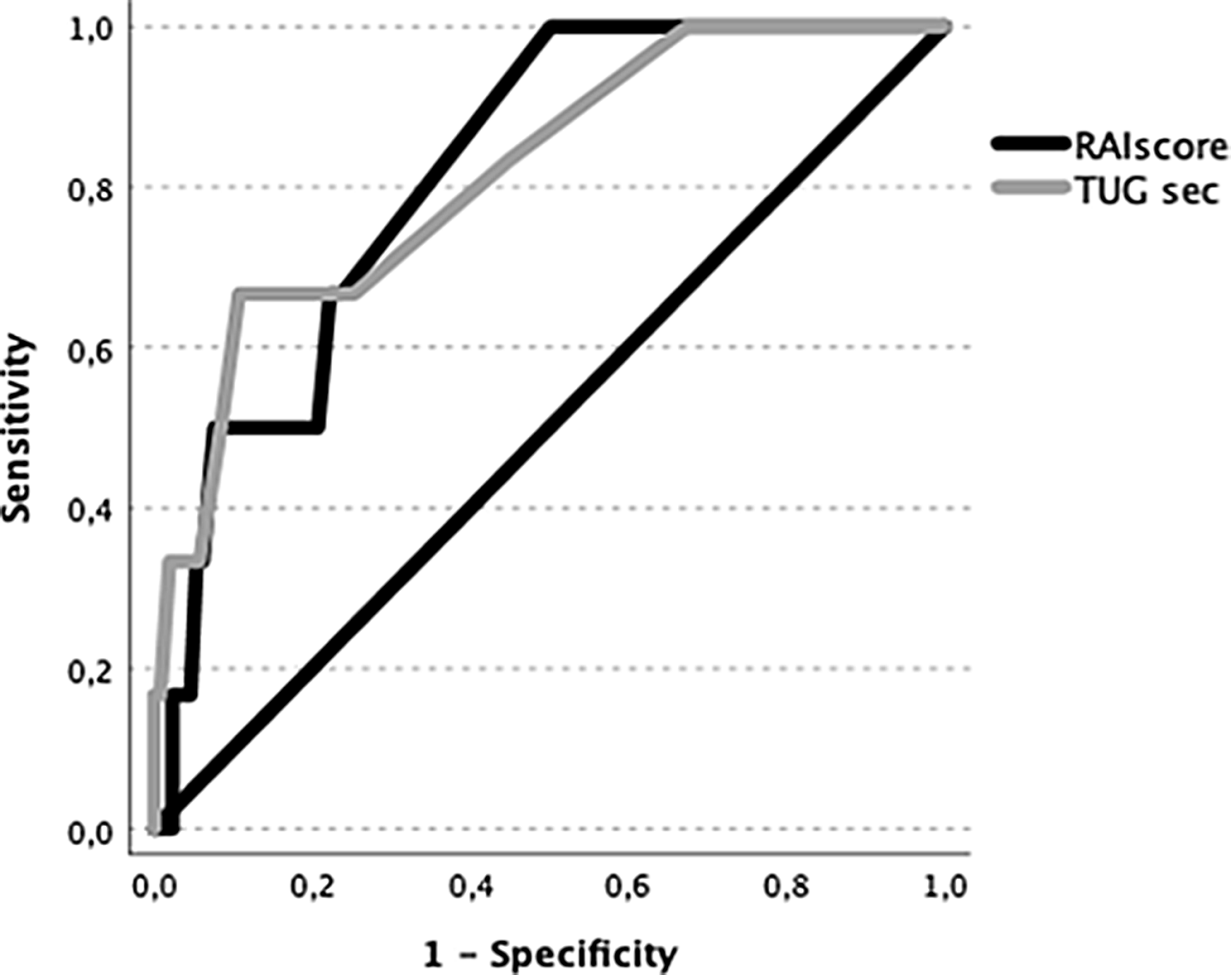
Receiver Operator Curves (ROC) for the Timed Up and Go Test (TUG) and the Risk Analysis Index (RAI score). The area under the curve (AU)-ROC for the TUG was 0.818, with a predictive cut-off of 8 s (Sensitivity 75%, specificity: 65%, *p* < 0.001). The AU-ROC for the RAI scoring was 0.804, with a predictive cut-off of 25 (Sensitivity 83%, specificity 62%, *p* < 0.001)

Additional ROC analyses were performed to detect TUG and RAI scoring cut-off values. Both analyses revealed a highly correlative area under the curve > 0.8 for 90-day survival. The AUROC for the TUG was 0.818, with a predictive cut-off of 8 s (Sensitivity 75%, specificity: 65%, *p* < 0.001). The AUROC for the RAI scoring was 0.804, with a predictive cut-off of 25 (Sensitivity 83%, specificity 62%, *p* < 0.001). The results are displayed in Fig. 1.

Univariate and multivariate analyses were performed to identify independent risk factors for postoperative mortality, complications in general and overall complications. Risk factors for postoperative mortality from univariate analysis depicted by the p-values from X^2^-test results were the RAI-risk score (p < 0.001), the TUG-risk score (p < 0.001), the total risk score (p = 0.04) and the activities of daily living ‘toilet use’ (p < 0.001) and ‘mobility’ (*p* < 0.009).

Risk factors for major complications (CD > 3) were anemia (*p* = 0.01), malignant diagnosis (*p* = 0.02), the type of the procedure (*p* = 0.004), the RAI-risk score (*p* = 0.02), the TUG-risk score (0.009), the total risk-score (*p* = 0.001), and weight loss (*p* = 0.002).

Risk factors for any complication CD 1 to 5 were a malignant diagnosis (*p* = 0.08), the type of procedure (*p* = 0.002), sarcopenia (*p* = 0.01), and weight loss (*p* = 0.002). In the multivariate analyses, none of the factors entered the logistic regression model successfully. Data are displayed in Table 3.

### Performance of the risk groups

**Fig. 2:**
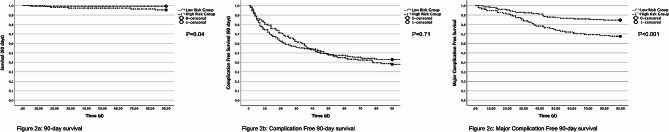
**a-c** 90-day survival (a), complication-free 90-day survival (b), and major complication-free 90-day survival (c) comparing high vs. low-risk groups

Importantly the risk grouping was set up before the study was launched within the app and was based on evidence from the literature. The low-risk group consisted of 155 patients and the high-risk group of 112 patients. Kaplan-Meier curves for 90-day overall survival (low risk: 99.4% vs. high risk: 95.5%; p = 0.04) and the occurrence of major complications (low risk: 16.4% vs. 32.4%; p < 0.001) showed a significant difference between the two risk groups favoring the low-risk group as predicted. Both groups were predictive for mortality 90 days after surgery as outlined above. Interestingly, the occurrence of any complication in the two groups was not significantly different (low risk: 57.3% vs. 63.1%; 0.71). These findings support the ‘failure to rescue theory’ in vulnerable cohorts and require special attention. Data are shown in Fig. 2a-c. Interestingly, the Kaplan-Meier analysis of sarcopenia as a discriminating risk factor did not lead to significantly different results (90-day survival: sarcopenic: 96.3% vs. non-sarcopenic: 96.8%; *p* = 0.92. Any complication: 75.6% vs. 51.6%; *p* = 0.08). Major complications: 25.6% vs. 12.9%; *p* = 0.19). Here the availability of only 113 data sets very likely impairs the findings, as there is a huge trend concerning the prevalence of any complication in the presence of sarcopenia; however, not statistically significant.

## Discussion

The screening of patients before major abdominal and thorax surgeries with ‘The Prehab App’ risk calculator has proven to correlate significantly with the short-term surgical quality reflected by 90-day survival rates. The primary intention of being able to estimate the risk for high- and low-risk patients was met in this clinical trial. The specific evidence-based tools, like the timed up-and-go test and the risk analysis index, correlated well with 90-day survival. They delivered significant cut-offs for this endpoint in the receiver operator curve analysis. To our knowledge, this is the first European cohort that underwent validation of risk scoring with the risk analysis index in general and the first time in a digitized tool globally [8].

The arguments highlighting the importance of baseline risk assessment before major surgeries are multiple. First, the analysis and identification of pretherapeutic risk factors help in the risk-benefit assessment for every patient. Second, the modifiable risk factors may be approached by prehabilitation, a combination of steered physical activity, nutritional advice, and mental wellbeing. A set of risk scores is needed to measure the benefit of prehabilitation. The RAI score is a prospective score for surgical patients with a sensitivity of 0.5 and a specificity of 0.82 in predicting 180-day mortality [6, 29, 30]. Fourteen items result in a score of 0 to 81; higher scores show higher frailty [31]. It consists of age, clinical risk factors, and modified ADL and has been validated in millions of patients. It correlates highly with failure to rescue after major surgery with rising scores [23, 30, 31]. The RAI-C score has been validated prospectively in a cohort of 6.856 patients, representing an effective tool to measure frailty-associated risk in patients before surgery with a high predictive value for 180-day mortality (C-statistics 0.77) [6]. Another prospective study, including 984.550 patients stratified into five different RAI-C-based risk groups, revealed a dose-response association between frailty and postoperative complications and displayed a convincing prediction of failure to rescue, which has been present in the here published high-risk group as well [23].

Similarly, the ECOG performance status demonstrated a highly predictive clinical score that was validated in two studies of patients with ovarian cancer undergoing cytoreductive surgery, exhibiting that ECOG > 0 or 1 significantly correlated with severe postoperative complications (OR 13.3, *p* = 0.01) [32, 33]. The ECOG score also independently associated with 30-day mortality in patients undergoing high-risk emergency laparotomy (OR 5.9, *p* < 0.01) [34] and showed a better discrimination capacity of 30-day mortality than the Charlson comorbidity index in a study with 327 patients undergoing non-cardiac surgeries (ECOG: 0.98, *p* < 0.001; mFI-5: 0.86, *p* < 0.001; Charlson Score: 0.53, *p* = 0.71; fall risk assessment: 0.55, *p* = 0.44) [35]. A third widely used risk scoring system is the Timed Up and Go Test (TUG). Research shows that the TUG is a valid predictor for increased postoperative mortality [24].

Stakeholders in healthcare are eager to work with assessment tools to steer quality to easily document their own outcomes and to compete with benchmarking data rpovided by third parties. Most of the stakeholders, no matter if in the hospital management or front line staff caring for patients have a lack of instruments and wait for well designed digital solutions as our group under the lead of Dora Zmuc found out in two different surveys performed in Germany and Slovenia in 2021 [36].

By setting up the app there was a profound discussion to use evidence-based tools that were easily to use without any further aids that could be a barrier. For example the American College for Surgeons NSQIP (National Surgical Quality Improvement Program) published and validated by Bilimoria more than 10 years ago is a very refined risk calculator for surgical patients but does not meet the requirements for detecting the risk for a patient that should undergo prehabilitation in a highly elective surgical indication [37]. This means that it requires more ressources to collect and enter the data into the calculator. Furthermore, it lacks prove of general transferability to Germany or a European setting [38, 39]. The assessment with The Prehab App risk calculator did take less than 5 min before the surgical procedure. The follow up data with including diagnose, procdeure and the most severe complication using the Dindo Cavien score instead of the cumulative comprehensive complication index simplifies the assessment by nonetheless high validity of the DIndo Clavien score [20, 21, 40, 41].

In general, The Prehab App provides a novel approach for an individual endurance exercise program based on a structured risk assessment, incorporating a set of validated measures (including the RAI-scoring, ECOG, Timed Up-and-Go, and hemoglobin) and stratifying patients into specific risk groups for prehabilitation. A variety of risk assessment tools in the doctors’ version tested here, have the potential to efficiently asses the patients’ risk factors before surgery and predict postoperative outcomes [6, 23, 32, 33]. To apply to everyday clinical work, they need to be used promptly, without any extra tools (like grip strength measuring tools that can never be found in outpatient care as they may be in the pocket of a colleague who is on holiday), be easily integrated into taking the patient’s history and preoperative workup, and should consist of data that are globally available in every patient [8].

The presented complication rates are similar to the initial description of the Clavien-Dindo classification of complications by Dindo et al. [20]. The overall complication rates were similar between the different specialties and displayed the usefulness and repeated validation in major surgery as approached with The Prehab App Doctors’ version. As a limitation, one could judge the fact that we did not include patients undergoing orthopedic or cardiac surgery. Those patients often have contraindications against a potentially unsupervised moderate to vigorous aerobic exercise program or physical limitations requiring certain muscle strengthening exercises or refraining from interval training. The evidence generated by this prospective trial is limited to one center. However, with the contribution of multiple independent acting departments within a major tertiary care center and University Hospital as well as a comprehensive cancer center in Germany, the study became an institutional collaborative approach.

The results of this study will be used to adjust the software and comply with the MDR’s requirements and regulations for certification. This includes adaption of the risk management, and shaping of the intended use, as the risk calculator could be used sufficiently in all abdominal and thoracic surgical specialties performing major surgeries [28]. In the next step, the risk assessment in combination with the automatically created 3 to 6-week exercising program will be explored in a randomized-controlled safety and efficacy study comparing patients with a tailored and digitized app-based prehabilitation program vs. patients with no specific structured program to exercise, nutritional support or mental wellbeing. The control group, however, will receive standardized information about the items of prehabilitation, and a recording of the performed physical activity will be performed. Considering the limitations of the trial, especially by a lack of predictive factors from the multivariable trial, the number of patients for a deeper analysis was probably too low. However, this was not the primary aim in this clinical investigation and must be tested at a later stage in a real-life cohort. Furthermore, the distribution of female participants of roughly one third may bias the results. This will be one of the stratifications that we will approach in the randomized controlled trial currently prepared.

The pilot trial has considerable limitations that will be addressed in the following. Risk assessment is relevant and important for all surgical disciplines performing major surgeries. As this risk assessment is intended to define prehabilitation programs for patients that will undergo major surgery in the abdomen and in the thorax, there is a heterogeneity in indications that were considered in the risk management before. The approach was rather a top-down approach and apply a system that can be used easily by other disciplines (“use existing infrastructure”) rather than a bottom-up approach to design individual small risk calculators for every single discipline. This offers the chance to add digital modules to each discipline, like a pelvic floor risk assessment and prehab tool, a post hepatectomy complication tool kit or sarcopenia modulating tools. In this pilot trial it was furthermore difficult to perform meaningful deeper subgroup analysis (no differences were detected as outline in the supplementary material) or apply methods of artificial intelligence. The data generated here are more the basis for a future database that will be analyzed with predictive and generative artificial intelligence methods to predict individual risk and compare overall and subgroup outcomes.

Considering all the results shown here, the pilot trial indicated that the risk assessment may be viable, valid, and reliable method to identify risk factors before major surgeries. Risk groups (RAI, ECOG, TUG, and anemia) correlated well with postoperative mortality. Further studies have already been undertaken to evaluate the safety and validity of the app by comparing ECG data and data obtained by the app during individual aerobic exercise testing and are currently published by the group, including usability testing as required by regulatory bodies [42]. Nonetheless, especially nutritional assessment and a multicentric approach are required to put the evidence on a broader and more reliable basis.

## Conclusion

The proof-of-concept trial showed that a risk assessment with ‘The Prehab App’ may be viable to estimate the preoperative risk for mortality and major complications before major surgeries. The overall performance in this initial set of data indicated a certain reliability of the scoring and risk grouping, especially of the RAI score and the TUG. A larger multicentric data set will be required to proof the generalizability of the risk scoring to every subgroup and may be fostered by artificial intelligence approaches.

**Table 3:** Uni- and multivariable analysis of risk factors for 90-day survival, major complications, and any complications. Abbreviations: CD: Clavien-Dindo; HR: hazard ratio; CI: confidence interval; HR: heart rate; RAI: risk analysis index

Uni- & Multivariable Analysis	Univariable			Multivariable		
	Mortality (p-value from Pearson Chi^2^testing)	CD 3 to 5 (p-value from Pearson Chi^2^testing)	CD 1 to 5 (p-value from Pearson Chi^2^testing)	Mortality HR (95%CI; p)	CD 3 to 5 HR (95%CI; p)	CD 1 to 5 HR (95%CI; p)
Gender	0.39	0.58	0.65			
Smoker	0.98	0.49	0.74			
ECOG	0.16	0.78	0.19	0.64 (0.36 to 1.13; *p* = 0.12)		0.97 (0.83 to 1.12; *p* = 0.63)
Anemia	1.0	0.01	0.33		1.13 (0.80 to 1.57; *p* = 0.49)	
Sarcopenia	0.91	0.14	0.01			1.70 (0.94 to 3.10; *p* = 0.08)
HR changing medication	0.81	0.62	0.77			
Protein-enriched drinks	0.45	0.57	0.93			
Malignant disease	0.13	0.02	0.08	1.13 (0.55 to 2.06; *p* = 0.84)	1.14 (0.58 to 2.21; *p* = 0.70)	1.07 (0.54 to 2.08; *p* = 0.84)
Medical specialty	0.85	0.27	0.17			0.97 (0.83 to 1.12; *p* = 0.63)
Type of procedure	0.50	0.004	0.002		1.0 (0.99 to 1.01; *p* = 0.21)	1.00 (0.99 to 1.01; *p* = 0.34)
RAI score	< 0.001	0.02	0.20	3.45 (0.39 to 30.3; *p* = 0.26)	1.01 (0.61 to 1.93; *p* = 0.79)	1.11 (0.64 to 1.94; *p* = 0.70)
TUG	< 0.001	0.009	0.75	8.28 (0.82 to 84.1; *p* = 0.07)	1.01 (0.58 to 1.79; *p* = 0.17)	
High-risk patient	0.04	0.001	0.38	1.66 (0.09 to 30.3; *p* = 0.73)	0.77 (0.37 to 1.59; *p* = 0.48)	1.01 (0.69 to 1.46; *p* = 0.96)
Weight loss	0.81	0.002	002		1.29 (0.89 to 1.88; *p* = 0.17)	1.24 (0.84 to 1.82; *p* = 0.27)
Renal failure	0.59	0.97	0.73			
Cardiac failure	0.59	0.83	0.91			
Inappetence	0.15	0.69	0.21	1.29 (0.79 to 2.12; *p* = 0.31)		
Shortness of breath	0.83	0.35	0.77			
ADL mobility	0.009	0.38	0.68	1.72 (0.21 to 13.9; *p* = 0.61)		
ADL eat	0.99	0.62	0.93			
ADL toilet	< 0.001	0.56	0.86	0.53 (0.07 to 13.9; *p* = 0.61)		
Cognitive decline within three months before surgery	0.53	0.32	0.76			

### Electronic supplementary material

Below is the link to the electronic supplementary material.

Supplementary Material 1

## Data Availability

No datasets were generated or analysed during the current study.

## Abbreviations

ADL: Activities of Daily Living
ASA: American Society of Anesthesiologists
AUROC: Area Under the Curve Receiver Operator Characteristics
BfArM: Federal Institute for Pharmaceuticals and Medical Devices
BMI: Body Mass Index
Bpm: Beats per minute
CD: Clavien Dindo
CI: Confidence Interval
DIN: German Institute for Standardization
DRKS: German Clinical Trial Registry
ECOG: Eastern Cooperative Oncology Group
EN: European Norm
Eudamed: European Database on Medical Devices
F: Female
GI: Gastrointestinal
HgB: Hemoglobin
HPB: Hepatopancreatobiliary
HR: Hazard Ratio
HR: changing
Medication: Heart rate changing medication
ICD: International Classification of Diseases
IRB: Independent Review Board
ISO: International Standardization Organization
Kg: Kilogramm
M: Male
MDR: Medical Device Regulation
MPDG: Medical Device Implementation Act
OPS: Operating Procedures System
RAI: Risk Analysis Index
ROC: Receiver Operator Characteristics
SD: Standard Deviation
Sec: Seconds
SMA: Skeletal Muscle Area
SMI: Skeletal Muscle Index
STROCCS: Strengthening the Reporting of cohort, cross-sectional and case-control studies in Surgery
TUG: Timed Up and Go
Y: Year

## Data access

Data can be accessed anonymously on specific requests and the requesting group’s research question outline.
