# Supplementary material for: Predictive value of a novel digital risk calculator to determine early patient outcomes after major surgery: a proof-of-concept pilot study
Source: Patient Saf Surg. 2024 Apr 12;18:13. doi: 10.1186/s13037-024-00395-y (PMC11010393; doi:10.1186/s13037-024-00395-y)
Supplement: Supplementary file 1 — Supplementary Material 1 [file 13037_2024_395_MOESM1_ESM.docx]

**Supplementary tables and figure**

**Medical specialty and complications**


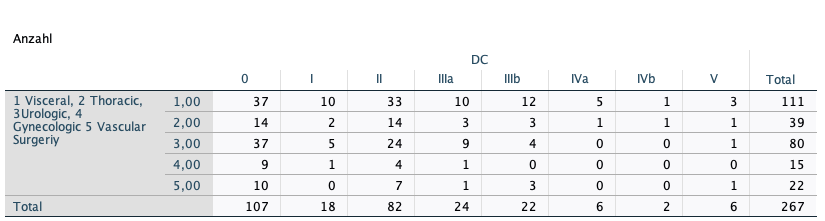


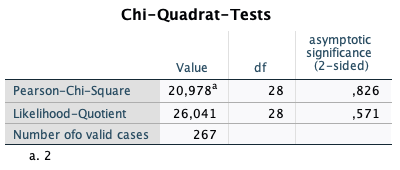


**Surgical procedure and complications**


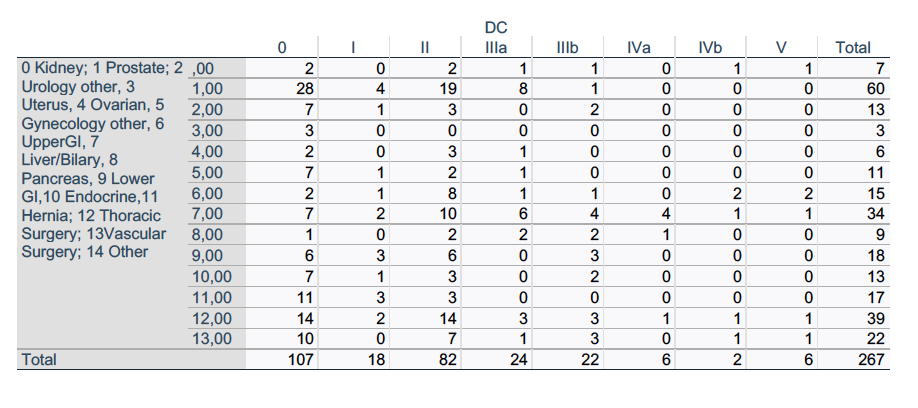


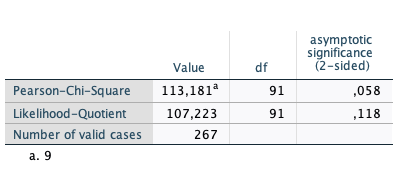


**Surgical procedure and any complication**


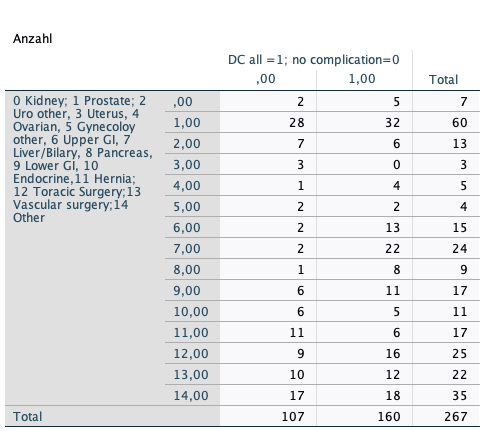


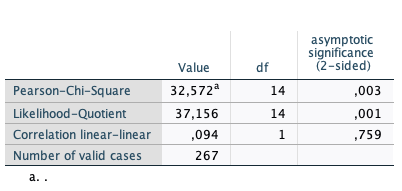


**Complication-free survival and speciality**


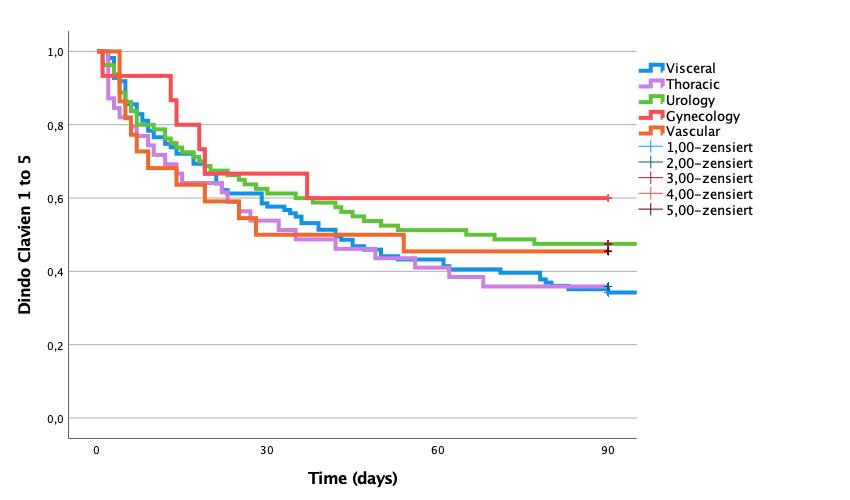


P=0.038
